# Impact of voluntary exercise and housing conditions on hippocampal glucocorticoid receptor, miR-124 and anxiety

**DOI:** 10.1186/s13041-015-0128-8

**Published:** 2015-07-02

**Authors:** Alejandro Pan-Vazquez, Natasha Rye, Mitra Ameri, Bethan McSparron, Gabriella Smallwood, Jordan Bickerdyke, Alex Rathbone, Federico Dajas-Bailador, Maria Toledo-Rodriguez

**Affiliations:** Queens Medical Centre, School of Life Sciences, University of Nottingham, Nottingham, NG7 2UH United Kingdom; Present address: MRC Centre for Developmental Neurobiology, Institute of Psychiatry, Psychology and Neuroscience, King’s College London, London, SE1 1UL United Kingdom

**Keywords:** Epigenetics, Glucocorticoid receptor, Exercise, Stress, microRNA

## Abstract

**Background:**

Lack of physical activity and increased levels of stress contribute to the development of multiple physical and mental disorders. An increasing number of studies relate voluntary exercise with greater resilience to psychological stress, a process that is highly regulated by the hypothalamic-pituitary-adrenal (HPA) axis. However, the molecular mechanisms underlying the beneficial effects of exercise on stress resilience are still poorly understood. Here we have studied the impact of long term exercise and housing conditions on: a) hippocampal expression of glucocorticoid receptor (*Nr3c1*), b) epigenetic regulation of *Nr3c1* (DNA methylation at the *Nr3c1-1F* promoter and miR-124 expression), c) anxiety (elevated plus maze, EPM), and d) adrenal gland weight and adrenocorticotropic hormone receptor (*Mc2r*) expression.

**Results:**

Exercise increased *Nr3c1* and *Nr3c1-1F* expression and decreased miR-124 levels in the hippocampus in single-housed mice, suggesting enhanced resilience to stress. The opposite was found for pair-housed animals. Bisulfite sequencing showed virtually no DNA methylation in the *Nr3c1-1F* promoter region. Single-housing increased the time spent on stretch attend postures. Exercise decreased the time spent at the open arms of the EPM, however, the mobility of the exercise groups was significantly lower. Exercise had opposite effects on the adrenal gland weight of single and pair-housed mice, while it had no effect on adrenal *Mc2r* expression.

**Conclusions:**

These results suggest that exercise exerts a positive impact on stress resilience in single-housed mice that could be mediated by decreasing miR-124 and increasing *Nr3c1* expression in the hippocampus. However, pair-housing reverses these effects possibly due to stress from dominance disputes between pairs.

## Introduction

Lack of physical activity and increased levels of stress strongly contribute to the development of physical and mental disorders [1]. Psychological stress has been associated with multiple medical conditions, from coronary heart disease [2] to depression [3]. Conversely, exercise has been shown to improve and prevent both physical and mental diseases [4–6] and thus it is increasingly used for the treatment and prevention of mental disorders [7]. There are numerous advantages when using exercise to improve mental health, such as cost-effectiveness, negligible side effects, additional health benefits and sustainability after the end of the treatment.

The mechanisms underlying the mental health benefits of physical exercise are thought to be related, in part, to reduced levels of anxiety and increased stress coping ability [8]. Nonetheless, the molecular processes mediating the beneficial effects of exercise on stress remain unclear. The glucocorticoid receptor (GR) could play a very important role. Both, stress and exercise increase the activation of the hypothalamic-pituitary-adrenal axis (HPA axis), which is regulated by glucocorticoids via a negative feedback loop [9]. However, stress and exercise differ on the timing and strength of HPA activation and therefore on the consequent impact on mental health. While exercise produces an acute increase of circulating glucocorticoids, stress leads to chronic activation of the HPA axis. Indeed the acute elevations in glucocorticoids linked to exercise seem to have a protective effect against some of the adverse consequences of chronic exposure to high glucocorticoid concentrations, such as insulin resistance and development of diabetes [10].

Glucocorticoids maintain HPA homeostasis after stress via activation of GR in the hippocampus. Increased GR expression at the hippocampus is highly correlated to enhanced resilience to stress [11, 12]. Conversely removal of circulating glucocorticoids increases GR expression in the hippocampus [13]. Thus, we hypothesized that the beneficial effects of exercise on stress resilience might be related to increased expression of hippocampal GR.

While the GR is widely expressed throughout the body, its expression is tightly regulated in different tissues and developmental stages [12, 14]. This regulation can happen at the transcriptional or translational level [15]. There are multiple splice variants of the GR gene (*Nr3c1*) resulting from alternative first exon usage [16]. The promoters for many of these alternative first exons lie in regions with high CpG content (CpG islands). Therefore their transcription can be modulated via changes in DNA methylation [17]. In the rat, it has been shown that early life events have significant impact on gene expression and DNA methylation of *Nr3c1*, in particular the hippocampus-specific exon 1.7 (exon 1 F in mouse and human) [18, 19].

A second layer of regulation of *Nr3c1* expression is via RNA silencing by microRNAs. The 3′ untranslated region of the *Nr3c1* gene contains multiple microRNAs’ seed regions, including miR-124 and miR-18 [20]. While miR-18 is expressed in multiple tissues, miR-124 is particularly enriched in the brain [21] and has been shown to inhibit *Nr3c1* expression in cultured cells and *in vivo* [22]. Interestingly, miR-124 expression in the hippocampus peaks during the stress hyporesponsive period, when mild stressors do not produce elevation in glucocorticoid levels in neonates [22].

A third level of regulation of *Nr3c1* expression comes from the adrenal gland where glucocorticoids are produced in response to activation of the adrenocorticotropic hormone receptor (ACTH-R, also known as melanocortin 2 receptor, MC2R). Physical exercise acutely activates the adrenal gland resulting in increased release of glucocorticoids. Long-term physical activity produces biochemical changes and hypertrophy in the adrenal gland [23, 24], which is also observed in chronic stress [25].

It is widely believed that, for young rodents, group-housing is less stressful than single-housing. For example, post-weaning isolation rearing is a preclinical rodent model for schizophrenia [26]. However, the impact of single-housing, particularly in mice, differs when animals are single-housed in adulthood [27, 28]. Additionally, for male mice pair-housing seems to be a more stressful experience than group-housing (3 or more animals per cage), particularly when the mice do not belong to the same litter, since they fight for dominance [29, 30]. For example learning is affected by the acquisition of dominance status in pair-housed male mice [31]. Conversely environmental enrichment, where animals are housed in larger groups with toys and running wheels, has been shown to be beneficial for multiple conditions (e.g. brain injury, ageing, prenatal alcohol exposure) [32]. However it seems that many of the positive effects of environmental enrichment depend on the mice getting free access to running wheels [33, 32].

These observations have led us to hypothesize that long-term exercise could improve resilience to stress through increased hippocampal *Nr3c1*, which might be mediated via epigenetic modifications. Thus, the aim of this study was to investigate the impact of exercise and housing conditions on: a) *Nr3c1* expression in the hippocampus, in particular *Nr3c1-1F*, b) *Nr3c1* epigenetic regulation (miR-124 expression and DNA methylation at the promoter region of *Nr3c1-1F*), c) anxiety and d) adrenal gland weight and *Mc2r* expression.

## Results & discussion

Using a rodent model of long-term voluntary exercise we studied, at the molecular level, the mechanisms by which exercise improves mental health.

### Distance run and impact of housing and exercise on weight gain and food intake

**Fig. 1:**
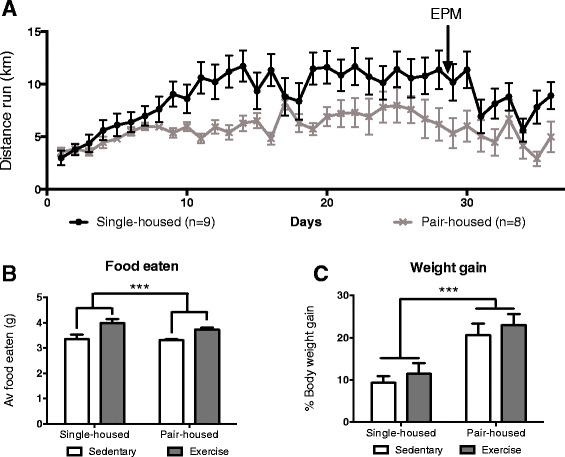
Distance run and impact on body weight and food consumption. **a** Daily distance run by single-housed and pair-housed mice during the studies. Arrows indicate the dates when the behavioural tests were performed. **b** Average food consumed and **c** weight gained during the study. *** *p* < 0.001

Both, single-housed and pair-housed animals spontaneously started running immediately after getting access to the wheels, increasing their daily running distance over time. During the first week of free access to a running wheel, single-housed and pair-housed mice ran similar daily distances (Fig. 1a), starting from 2.6 to 3 km/day and steadily increasing in both groups. This is in agreement with findings from previous studies for single-housed mice [34]. However, after the first week, pair-housed mice slowed their increase in daily running and ended up running significantly shorter distances than single-housed mice (reaching daily running distances of 6–8 km/day for pair-housed mice versus 10–12 km/day for single-housed mice) [*t* (70) = −5.916, *p* < 0.0001]. This difference was not due to lack of opportunity to run on the wheel as the cages of the pair-housed mice contained two wheels, so that, both mice could run simultaneously. Thus the lower daily running distance when pair-housed, was likely due to the dominant mouse fighting the submissive mouse over the use of the wheels. To confirm this, future experiments should study the behaviour of the mice during the dark/active period (when the animals run).

Animals that exercised ate significantly more during the study, without any significant increase in body weight [*F* (1,25) = 19.896 *p* < 0.000] (Fig. 1b). Indeed there was a significant correlation between food eaten and distance run [Pearson’s r (17) =0.706, *p* = 0.002]. Housing had a significant effect on body weight increase during the study (pair-housed mice showed a higher increase in body weight) [*F* (1,25) = 15.023 *p* = 0.001] (Fig. 1c).

### The effect of exercise on hippocampal *Nr3c1* expression differs depending on the housing conditions

**Fig. 2:**
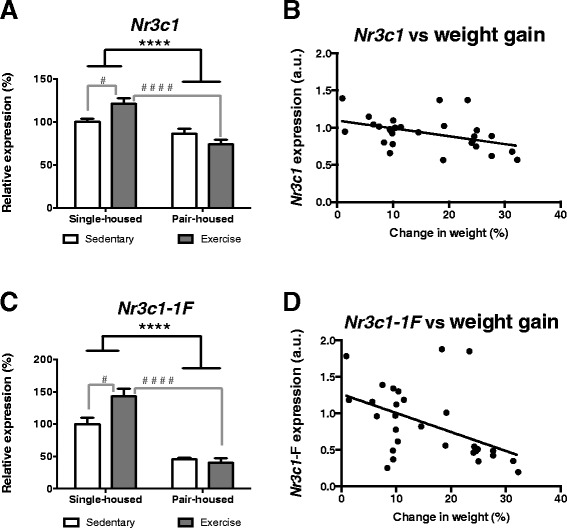
Impact of exercise and housing conditions on *Nr3c1* and *Nr3c1-1F* expression at the hippocampus. **a** *Nr3c1* and **c** *Nr3c1-1F* expression for single-housed and pair-housed mice that exercised or remained sedentary. Pearson’s correlation between weight gain and hippocampal expression of **b** *Nr3c1* or **d** *Nr3c1-1F*. **** *p* < 0.0001 housing effect; # *p* < 0.05 post hoc comparison between single-housed mice that exercised and sedentary single-housed mice; # # # # *p* < 0.0001 post hoc comparison between mice that exercise single-housed and pair-housed

Animals pair-housed expressed lower levels of glucocorticoid receptor mRNA (*Nr3c1*) in their hippocampus [Two-way ANOVA, *F* (1,25) = 29.90 *p* < 0.0001] (Fig. 2a). Moreover a significant housing*exercise interaction was observed [*F* (1,25) = 9.07 *p* = 0.006]. Post hoc comparisons indicated that single-housed mice that exercised had significantly higher *Nr3c1* expression than single-housed sedentary mice [*t* (12.75) = 2.946 p = 0.012] (Fig. 2a) and pair-housed mice that exercised *t* (13) = −5.464 *p* < 0.0001] (Fig. 2a). We also found a significant negative correlation between the weight gained during the experiment and *Nr3c1* expression in the hippocampus [Pearson’s r (29) = −0.440, p = 0.0170] (Fig. 2b). These results suggest that exercise might increase psychological resilience in single-housed mice (via an increase in hippocampal *Nr3c1* expression) while pair-housing might be more stressful than single-housing. This is the first time an increase in *Nr3c1* as result of exercise is reported in the hippocampus. Moreover, our results are in agreement with previous observations that exercise prevented the decrease in glucocorticoid receptor in the hippocampus in single-housed Zucker diabetic fatty rats [35]. As *Nr3c1* is a transcription factor, changes in *Nr3c1* expression will probably affect expression of *Nr3c1* responsive genes. *Nr3c1* belongs to the family of ligand regulated nuclear receptors. Upon binding to glucocorticoids *Nr3c1* becomes activated, translocates into the nucleus and binds to response elements in the promoter regions of genes. While direct binding of activated *Nr3c1* to the DNA stimulates gene transcription, *Nr3c1* can also inhibit gene expression via transrepression of other transcription factors [36]. The course of transcriptional activation/repression by *Nr3c1* is highly dynamic and varies depending on the length of glucocorticoid stimulation. Exercise produces acute increase in circulating glucocorticoids [37]. Acute activation of hippocampal *Nr3c1* by glucocorticoids results in consecutive waves of gene expression [38]. One hour after activation there is downregulation of gene expression (e.g. mineralocorticoid receptor and monoamine oxidase A), while 3 h after activation there is upregulation (e.g. corticotropin-releasing-hormone receptor 1 and apolipoprotein E) and downregulation (e.g. monoamine oxidase A) of gene expression. Thus, in single housed animals, increased levels of *Nr3c1* in the hippocampus, combined with the exercise-induced acute increase of glucocorticoids might augment the availability of serotonin, dopamine and noradrenaline while increasing the effects of corticotropin releasing hormone in the hippocampus. In pair-housed animals the effects might be reversed due to decreased *Nr3c1* levels.

### Exercise increases *Nr3c1-1F* expression at the hippocampus without changing DNA methylation at its promoter

The *Nr3c1* gene contains multiple alternative first promoters where transcription can start. We focused on the hippocampus-specific promoter 1 F, which is involved in the regulation of *Nr3c1* expression by environmental factors [16, 39]. In agreement with our *Nr3c1* findings pair-housed animals expressed lower levels of *Nr3c1-1F* mRNA in their hippocampus [Two-way ANOVA, *F* (1,25) = 65.95 *p* < 0.0001] (Fig. 2c). Moreover, a significant housing*exercise interaction was observed [*F* (1,25) = 6.124 p = 0.020]. Post hoc comparisons indicated that: a) single-housed mice that exercised had significantly higher *Nr3c1-1F* expression than single-housed sedentary mice [*t* (14) = 2.577 p = 0.022] (Fig. 2c) and pair-housed mice that exercised [*t* (11.55) = −7.368 *p* < 0.0001] (Fig. 2c); while b) single-housed sedentary mice had significantly higher *Nr3c1-1F* expression than pair-housed sedentary mice [*t* (13) = −5.325 *p* < 0.0001] (Fig. 2c). This suggests that the effects of exercise upon *Nr3c1* expression are, at least in part, mediated by modulating the expression of the hippocampus-specific *Nr3c1-1F* splice variant. As for *Nr3c1* there was a significant negative correlation between the weight gained and *Nr3c1-1F* [Pearson’s r (29) = −0.482, p = 0.0081] expression in the hippocampus (Fig. 2d).

**Fig. 3:**
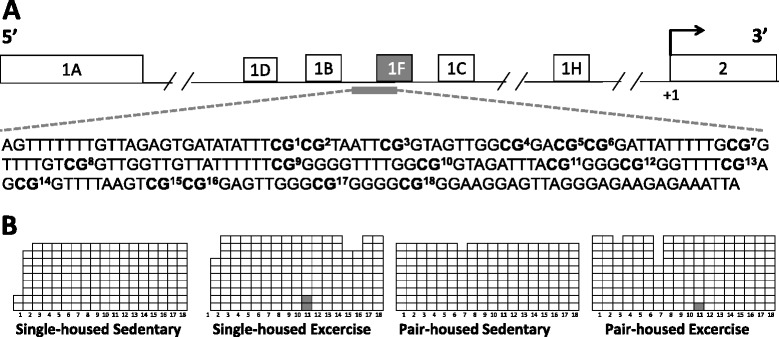
Impact of exercise and housing conditions on DNA methylation at the promoter region of *Nr3c1-1F*. **a** Schematic representation of the multiple first exons of the *Nr3c1* gene. +1 indicates the translational start site. The promoter region of *Nr3c1-1F* has been enlarged and the 18 CpGs it contains highlighted. **b** Methylation rates of *Nr3c1-1F* promoter are shown for single-housed mice and pair-housed that either exercised or remained sedentary. Each row represents a clone and each column indicates a CpG (empty square = not methylated CpG and full square = methylated CpG)

The promoter region of the *Nr3c1-1F* exon lies at a CpG island (Fig. 3a), therefore its transcription can be regulated by DNA methylation. Thus we studied whether the increase in *Nr3c1-1F* mRNA expression in mice that exercised was due to a decrease in DNA methylation. For this purpose we performed bisulfite sequencing of the proximal promoter region of *Nr3c1-1F*. We found virtually no DNA methylation at any of the 18 CpGs studied (Fig. 3b) although in CpG 11 exercise was associated with greater methylation [*X*^2^ (1) = 4.402, *p* = 0.036] (Fig. 3b). We can rule out the possibility that the lack of DNA methylation was due to technical problems, since we could detect full methylation when M.SssI methyltransferase was used to methylate the promoter region of *Nr3c1-1F in vitro* (data not shown). Interestingly, a similar lack of DNA methylation in the promoter region of *Nr3c1-1F* DNA at the hippocampus has been found in humans [40]. This might indicate that the promoter region of a gene with high expression demand, such as *Nr3c1,* needs to be demethylated to allow rapid transcription when needed.

### The effect of exercise on hippocampal miR-124 levels differs depending on the housing conditions

**Fig. 4:**
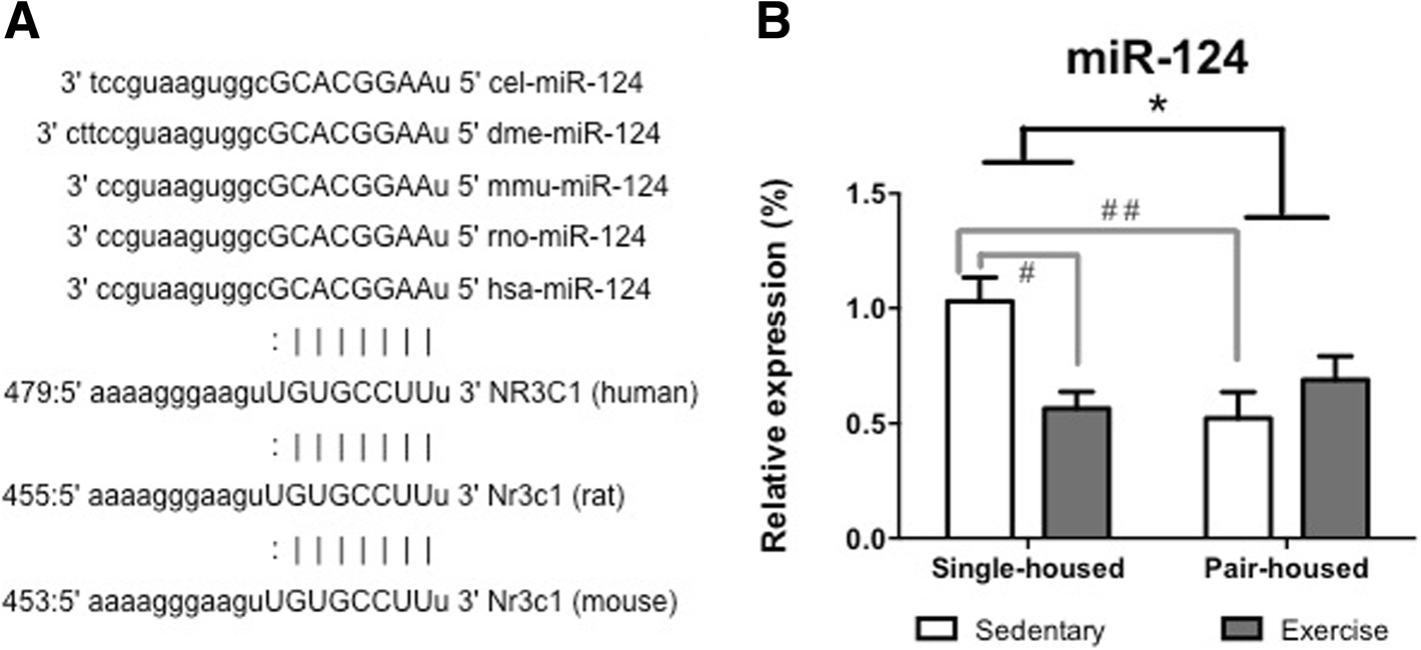
Impact of exercise and housing conditions on miR-124 levels at the hippocampus. **a** The mature sequence of miR-124 and the 3′ untranslated region of *Nr3c1* are highly conserved across species, suggesting a critical functional link between them (miRNA seed sequence shown in capitals). **b** Expression of miR-124 for single-housed mice and pair-housed that exercised or remained sedentary. * *p* < 0.05 housing effect; # *p* < 0.05 post hoc comparison between sedentary single-housed mice and single-housed mice that exercise; # # *p* < 0.01 post hoc comparison between sedentary single-housed mice and sedentary pair-housed mice

Another epigenetic mechanism that can regulate *Nr3c1* levels is silencing by non-coding RNAs. miR-124, a highly conserved microRNA (Fig. 4a) that is particularly enriched in the brain, was shown to repress *Nr3c1* expression both *in vitro* and *in vivo* [22]. Thus we studied whether exercise and/or housing conditions affected miR-124 expression in the hippocampus. We found that the effects of exercise and housing on miR-124 expression follow a pattern opposite to the one for *Nr3c1*. Pair-housed animals expressed lower levels of miR-124 [Two-way ANOVA, *F* (1,25) = 4.604 *p* < 0.042] (Fig. 4b). Moreover there was a significant housing*exercise interaction [*F* (1,25) = 6.302 *p* = 0.019]. Post hoc comparisons indicated that sedentary single-housed mice had significantly higher miR-124 expression than mice that exercised [*t* (14) = 2.485 p = 0.026] (Fig. 4b) and sedentary pair-housed mice [*t* (11) = −3.295 p = 0.007] (Fig. 4b). Additionally, *Nr3c1* and miR-124 levels were negatively correlated in the single-housed group [Pearson’s r (15) = −0.525, p = 0.045] but not in the pair-housed group [Pearson’s r (11) = 0.046, p = 0.892]. Since miR-124 has previously been shown to regulate *Nr3c1* [22], it could be hypothesized that the negative correlation between *Nr3c1* and miR-124 levels is due to a direct effect of decreased miRNA-124 on *Nr3c1* expression. However, further experiments are needed in order to validate whether this is a direct causal relationship. Pair-housing reduced both *Nr3c1* and miR-124 expression in the hippocampus. This could suggest the involvement of alternative epigenetic mechanisms probably activated by stress linked to pair-housing. A possible mechanism could be decreased histone acetylation in the pair-housed group due to higher stress levels. For example chronic variable stress significantly decreases histone acetylation (H3k12Ac) in the hippocampus [41]. Moreover, treatment with the histone deacetylase (HDAC) inhibitor TSA significantly increased *Nr3c1* expression in the hippocampus [18].

We may hypothesize that the different effects of exercise on *Nr3c1* and miR-124 expression in single versus pair-housed animals could be due to differences in pulsatile/continuous levels of corticosterone and distance run. As mentioned previously, exercise increases glucocorticoids in a pulsatile manner, while stress leads to overall increase of glucocorticoid levels. This could lead to different effects of housing on *Nr3c1* and miR-124 as, in the hippocampus, transient release of glucocorticoids promotes neurogenesis, learning and memory [42, 43]. Conversely prolonged secretion of glucocorticoids during chronic stress has the opposite effect [44, 45]. Finally, despite having two wheels per cage, pair-housed animals run approximately half the distance than the single-housed. This difference in distance run could also have affected expression levels. Future studies should investigate the role of circadian glucocorticoid release on exercise-induced changes of *Nr3c1* expression.

### Impact of exercise and housing on anxiety

Higher hippocampal levels of *Nr3c1* are correlated with a better ability to cope with stress, [11, 12]. Thus, we studied the impact of exercise and housing on anxiety using the EPM test. The EPM is widely used to study anxiety when the animals are exposed to unfamiliar, open and elevated locations. However, some of the EPM measures might be influenced by external factors (such as time of testing or prior exposure to behavioural tests). Thus, in order to reduce the chances that our results could be affected by differences in the motility of the animals (due to exercise-induced fatigue), we used ethological measures (stretch attend postures and unprotected head dips) as well as the conventional ratio “time in the open arms vs. all arms”.

**Fig. 5:**
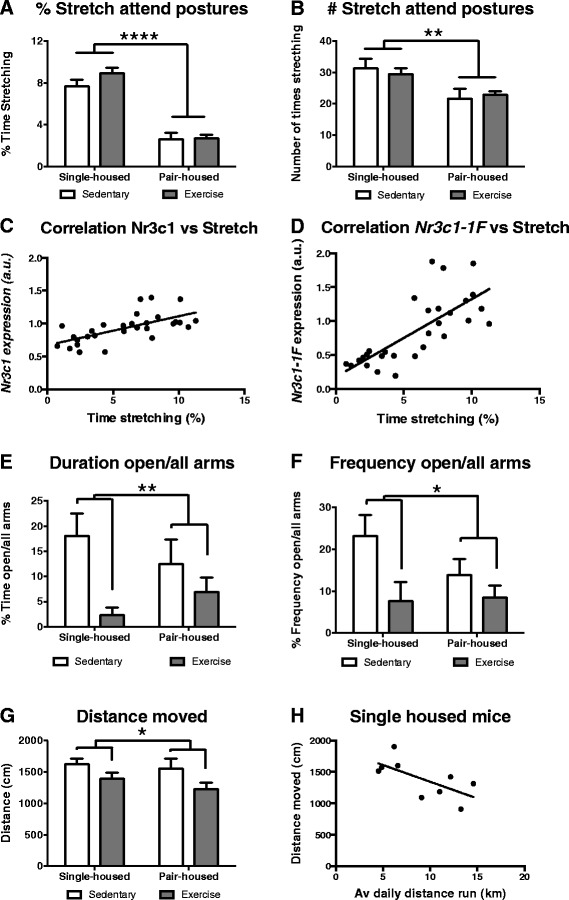
Impact of exercise and housing conditions on behaviour at the EPM test. Behavioural measures in the EPM test for single-housed mice and pair-housed that either exercised or remained sedentary. **a** Percentage of time and **b** frequency performing stretch attend postures. Pearson’s correlation between time performing stretch attend postures and hippocampal expression of **c** *Nr3c1* or **d** *Nrc31-1 F*. **e** Ratio time spent in the open arms versus all arms. **f** Frequency crossing into the open arms vs. all arms. **g** Distance moved. **h** Pearson’s correlation between distance moved in the EPM and daily distance run on the wheels. * *p* < 0.05 and ** *p* < 0.01

Stretch attend postures are ethologically relevant risk assessment behaviours displayed in potentially threatening situations [46, 47] and are correlated with corticosterone response [48]. Two-way ANOVA revealed that single-housed mice spent significantly more time stretching [*F* (1,28) = 111.4 *p* < 0.0001] (Fig. 5a) and stretched more times [*F* (1,28) = 11.59 p = 0.002] (Fig. 5b) from the central square into the open arms. Interestingly there was a significant positive correlation between the amount of time spent on stretch attend postures at the EPM and *Nr3c1* [Pearson’s r (29) =0.642, p = 0.0002] (Fig. 5c) or *Nr3c1-1F* [Pearson’s r (29) = 0.745, *p* < 0.0001] (Fig. 5d) expression in the hippocampus. There were no significant differences in the number of unprotected head dips over the edge of the open arm or time spent head dipping. The exercise-induced up-regulation in *Nr3c1* expression together with the higher percentage in stretch attend postures we found in single housed mice, further suggests that exercise might increase their resilience to stress. Conversely our results seem to indicate that pair-housing can be a stressful experience, which worsens when the animals have access to running wheels. Indeed, while individual housing of mice, particularly in young age, is usually thought to have a general detrimental effect, several studies have reported that single-housed mice show no change or even have decreased anxiety and reactivity to stressors [49]. Moreover previous studies have shown that, for male mice, pair-housing seems to be a more stressful experience than group-housing (3 or more animals per cage), particularly when the mice do not belong to the same litter, since they fight for dominance [29, 30].

Physical exercise led to a significant decrease in the percentage of time spent at the open arms versus all arms [*F* (1,28) = 9.251 *p* = 0.005] (Fig. 5e) and the frequency of entrance to the open arms versus all arms [*F* (1,28) = 6.331 *p* = 0.017] (Fig. 5f). No housing differences or housing*exercise interactions were found in these tests. These results are in agreement with Fuss et al. [50, 34] and Onksen et al. [51] who reported a decrease on the time spent in the open arms in animals that exercised. Interestingly the decrease in open arm entry/time by exercise was mechanistically related to hippocampal neurogenesis [50, 34, 51]. The significant decrease in the open/all arms ratio would suggest that exercise increases anxiety. However, based on previously published literature and motility at the EPM (see below), we could argue that performing the EPM during the light phase of the light/dark cycle might not be a good measure of anxiety when mice run 4–10 km/night (as the motility and tendency of the animal to move to open spaces might be affected by its physical activity during the night). Indeed Santos-Soto et al.*,* using a protocol similar to ours, but performing the EPM test during the first hour of the dark phase (active period for the mouse), reported that exercise significantly increased the ratio of time open/all arms [52]. Likewise, similar results were found for the rat when the EPM test was performed during the dark period [53]. It could be argued, as critically reviewed in [54], that despite its multiple positive outcomes, wheel running has also some potential negative side effects, which might affect the behaviour of the animal at the EPM. For example the tail of the rodent can get bent upwards, particularly when the animals have access to the wheel for extended periods of time (8 weeks or more [54]). As rodents require their tail for balance, this physical transformation can affect the performance of the rodents on the open arms of the EPM, where good balance is required. However none of our mice developed an “upward bent” tail, probably due to the fact that the surface of our running wheels was made of a continuous plastic sheet and the mice run for less that 5 weeks. Furthermore, our results show that the distance moved [*F* (1,28) = 5.79 *p* = 0.023] (Fig. 5g) and speed [*F* (1,28) = 6.110 *p* = 0.020] in the EPM was also significantly lower in the exercise group. Indeed, for single-housed mice that exercised, there was a significant negative correlation between the average distance run in the wheels and the speed [Pearson’s r (9) = 0.667, *p* = 0.049] and distance walked on the EPM [Pearson’s r (9) = 0.676, *p* = 0 .045] (Fig. 5h). Furthermore, as mentioned before, the percentage of time the mice spent on stretch attend postures, a sign of low stress levels, shows a strong tendency towards higher time stretching in single-housed mice that exercised.

### Impact of exercise and housing on adrenal weight and *Mc2r* expression

**Fig. 6:**
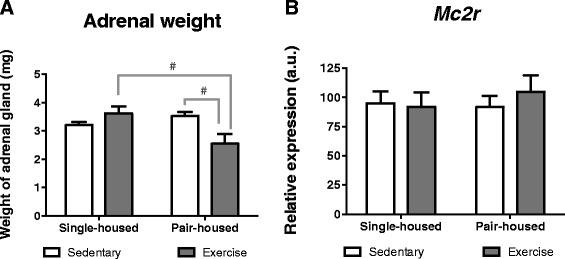
Effect of exercise and housing conditions on adrenal gland weight and adrenal *Mc2r* expression. Expression for single-housed mice and pair-housed that either exercised or remained sedentary. **a** Adrenal gland weight and **b** *Mc2r* expression. # *p* < 0.05

The adrenal gland is a stress responsive organ. While it is involved in the response to stressful stimulus (as part of the HPA axis), it is also affected by chronic stress (which usually increases adrenal weight) [25]. Thus, we determined whether housing or exercise had any effect on adrenal gland weight and/or adrenocorticotropic hormone receptor (*Mr2c*) expression at the adrenal glands. We found that exercise has opposite effects on adrenal weight depending on housing conditions. Two-way ANOVA showed a significant housing*exercise interaction for the adrenal gland weight [*F* (1,26) = 8.921 p = 0.006]. Post hoc comparisons indicated that pair-housed mice that exercised had significantly lighter adrenals than sedentary pair-housed mice [*t* (12.75) = −2.571 *p* = 0.033] (Fig. 6a) and single-housed mice that exercised [*t* (14) = −2.648 *p* = 0.019] (Fig. 6a). The exercise-induced increase in adrenal weight in single-housed mice is probably due to physical activity, as exercise leads to an acute increase in corticosterone. This could be beneficial, as previous studies have suggested that the increase in adrenal size in response to exercise is related to a higher sensitivity to changes in the HPA axis, producing faster decays in glucocorticoid levels in response to stress and ultimately improving stress resilience [55]. The increase in adrenal weight in the sedentary pair-housed mice possibly results from the increase in stress due to pair-housing as suggested by the *Nr3c1* results. The decrease in adrenal weight in animals pair-housed that exercise seems more puzzling. We could hypothesize that it is due to adrenal “burnout” by excessive corticosterone release, resulting from the combined effects of exercise and stress. Finally we found that neither exercise nor housing affected *Mc2r* expression in the adrenal gland (Fig. 6b). This is in agreement with studies by Droste et al. in the rat showing that 4 weeks voluntary running resulted in increase hippocampal *Nr3c1* without change in ACTH response to stress [56, 57].

## Conclusion

In summary, we conclude that exercise in single-housed mice causes an upregulation of *Nr3c1* expression in the hippocampus and exerts a positive impact on stress resilience. This increase was correlated with a downregulation of miR-124, a known epigenetic regulator of *Nr3c1*. Conversely; pair-housing reverses these effects possibly due to increased dominance disputes over access to the wheels. Overall, the results presented here provide new information about the consequences of exercise and housing on HPA axis regulation and their potential impact in stress resilience.

## Methods

### Animals

33, C57BL/6 mice (8 weeks old, purchased from Charles River, UK) were randomly distributed into 4 groups: single-housed exercise (*n* = 9), single-housed sedentary (*n* = 7), pair-housed exercise (*n* = 8) and pair-housed sedentary (*n* = 8). Mice were on a 12 h light-dark cycle with food and water available *ad libitum*. Mice in the exercise group had free access to a running wheel for the entire duration of the study. Two running wheels were placed in the cages of pair-housed mice that exercised. Mice in the sedentary group were housed with a non-functional wheel to control for environmental enrichment. Distance and time run by each animal were recorded daily. After 4 weeks mice underwent behavioural tests. 5 weeks after the start of the experiment mice were humanely killed by cervical dislocation and their brains and adrenal glands extracted and immediately frozen.

All procedures and animal care were in accordance with the UK Animals (Scientific procedures) Act 1986 and with the ethics committee guidelines of the School of Life Sciences, University of Nottingham.

### Elevated plus maze (EPM)

Four weeks after the start of the experiment (i.e. being single/pair | having access, or not to a running wheel) mice were evaluated using the elevated plus-maze (EPM) test. The EPM consisted of two opposing closed arms (surrounded by high walls) and two open arms (36 × 6 cm) that extend from a central platform (6 × 6 cm) elevated 89 cm above the floor. The mice were placed individually on the central platform facing a closed arm and were allowed to freely explore the maze for 5 min. The behaviour of each mouse was monitored using a video camera, and the movements of the mice were automatically registered and analysed with a computerized tracking system (Ethovision 9, Noldus IT, The Netherlands). Entry into an arm was defined as entry of all four paws into the arm. Measurements included: total distance moved, speed, time spent in the open and closed arms, number of times the animal entered each type of arm, latency before entering an open arm and number of defecations. The mice’s behaviour was recorded and later scored with in-house produced behaviour observation software (Clicker v1.13) by an observer blind to the treatment (housing/exercise). Stretch attend posture from the central square into the open arms was defined as postures where both rear legs of the mouse remain in the central square and the forepaws go forward into the open arm. Unprotected head dip was defined as times when the head of the mouse (or nose of the mouse) went below the edge of the platform. Times were automatically transformed to percentage values.

### Gene expression profiling by Q-PCR

RNA was isolated with the AllPrep DNA/RNA/Protein mini Kit (Qiagen, Germany) following the manufacturer’s instructions. 500 ng of RNA were reverse transcribed with Superscript III (Life Technologies, UK) and 15-mer random primers (Sigma, UK). Q-PCR reactions were then performed in triplicates with the SensiMix Plus SYBR Green PCR kit (Bioline, UK) and a RotorGene 3000 cycler (Qiagen, Germany). PCR primers for *Nr3c1* [NM_008173.3] [forward primer 5′- AGG CCG CTC AGT GTT TTC TA-3′ and reverse primer 5′- TAC AGC TTC CAC ACG TCA GC-3′], *Nr3c1-1F* [XM_006525663.2] [forward primer 5′- AGC CAG GGA GAA GAG AAA CT -3′ and reverse primer 5′- TAC CAG GGG GAG CTA AGG AT -3′] and *Mc2r* transcript variants 1–4 [NM_001271716.1, NM_008560.3, NM_001271717.1, NM_001301372.1] [forward primer 5′- TGG CAG TTT TGA AAG CAC AG -3′ and reverse primer 5′- GCA ATG ACA GAC AGG CTG AA -3′] were designed with the Primer3 software (www.genome.wi.mit.edu/cgi-bin/primer/primer3.cgi). Primer’s efficiency and specificity were verified using melting curve analysis (single melt curve peak). Gene expression was normalized to the geometric average of two control genes (*S18* and *Hprt1*) according to the GeNorm normalization [58]. The relative expression levels of each mRNA were calculated using a modified 2 Delta-Delta-Ct algorithm [59].

### Bisulfite sequencing

Genomic DNA was isolated with the AllPrep DNA/RNA/Protein mini Kit (Qiagen, Germany) following the manufacturer’s instructions. gDNA from animals from the same group was pooled (adding 200 ng gDNA from each animal) and 500 ng of the pooled gDNA from each group was bisulfited with the EZ DNA methylation kit (ZYMO research, USA), according to the manufacturer’s protocol. Afterwards, the promoter of the gene encoding *Nr3c1-1F* was amplified (forward primer: 5′- AGTTTTTTTGTTAGAGTGATATATTT -3′ and reverse primer: 5′- ATTTCTTTAATTTCTCTTCTCCCTAACTC -3′), subcloned into the pGEM-T easy cloning vector (Promega, UK) and 10–15 clones per experimental group were sequenced as previously described in [60]. Primers were designed using “MethPrimer” (http://www.urogene.org/cgi-bin/methprimer/methprimer.cgi)

### miR-124 analysis

cDNA was synthesised from mature miRNAs using the miRCURY LNA™ Universal cDNA synthesis kit (Exiqon A/S, Denmark), which uses a poly-T primer. Samples were run in triplicate using primers for U6 and mmu-miR-124-3p (MIMAT0000134) (Exiqon A/S, Denmark). Q-PCR was undertaken using the ExiLENT SYBR® Green mastermix kit (Exiqon A/S, Denmark), and the Applied Biosystems 7900HT Fast thermocycler was used in standard mode using cycling parameters recommended by Exiqon. These commercial primers, cDNA synthesis and QPCR amplification kits are specifically optimised for miRNAs detection. Data was acquired using Applied Biosystems SDS2.3 programme. ROX™ (Life Technologies, UK) was used as a passive reference for normalising for non-PCR related fluorescence variations, and was incorporated by SDS2.3 to calculate Ct values. The Ct values were analysed using the comparative Ct method, with U6 as the endogenous control and single-housed and sedentary animals as the calibrator. Spike in samples containing UniSp6 were used as inter plate calibrators and variation of readings between replicate plates was <1.5 %.

#### Statistics

Data was analysed using two-way ANOVA. For single comparison between groups we used Student’s *t*-test. Pearson’s correlation was employed to study the relationship between gene expression, running, weight and/or behaviour. The frequency of methylated CpGs was examined using log-linear analysis. SPSS 22.0 (IBM) Statistical package was used for the analysis. Statistics reported in the text and figures represent the mean ± S.E.M. For all tests, null hypotheses were rejected at probability level of *p* < 0.05.
